# Spleen-Resident CD4^+^ and CD4^−^ CD8α^−^ Dendritic Cell Subsets Differ in Their Ability to Prime Invariant Natural Killer T Lymphocytes

**DOI:** 10.1371/journal.pone.0026919

**Published:** 2011-10-31

**Authors:** Emilie Bialecki, Elodie Macho Fernandez, Stoyan Ivanov, Christophe Paget, Josette Fontaine, Fabien Rodriguez, Luc Lebeau, Christophe Ehret, Benoit Frisch, François Trottein, Christelle Faveeuw

**Affiliations:** 1 Center for Infection and Immunity of Lille, Institut Pasteur de Lille, Lille, France; 2 Université Lille Nord de France, Lille, France; 3 Centre National de la Recherche Scientifique (CNRS), UMR 8204, Lille, France; 4 Institut National de la Santé et de la Recherche Médicale (Inserm), U1019, Lille, France; 5 Institut Fédératif de Recherche 142, Lille, France; 6 Laboratoire de Conception et Application des Molécules Bioactives, Faculté de Pharmacie, CNRS, UMR 7199/Université de Strasbourg, Illkirch, France; University of North Dakota, United States of America

## Abstract

One important function of conventional dendritic cells (cDC) is their high capacity to capture, process and present Ag to T lymphocytes. Mouse splenic cDC subtypes, including CD8α^+^ and CD8α^−^ cDC, are not identical in their Ag presenting and T cell priming functions. Surprisingly, few studies have reported functional differences between CD4^−^ and CD4^+^ CD8α^−^ cDC subsets. We show that, when loaded *in vitro* with OVA peptide or whole protein, and in steady-state conditions, splenic CD4^−^ and CD4^+^ cDC are equivalent in their capacity to prime and direct CD4^+^ and CD8^+^ T cell differentiation. In contrast, in response to α-galactosylceramide (α-GalCer), CD4^−^ and CD4^+^ cDC differentially activate invariant Natural Killer T (iNKT) cells, a population of lipid-reactive non-conventional T lymphocytes. Both cDC subsets equally take up α-GalCer *in vitro* and *in vivo* to stimulate the iNKT hybridoma DN32.D3, the activation of which depends solely on TCR triggering. On the other hand, and relative to their CD4^+^ counterparts, CD4^−^ cDC more efficiently stimulate primary iNKT cells, a phenomenon likely due to differential production of co-factors (including IL-12) by cDC. Our data reveal a novel functional difference between splenic CD4^+^ and CD4^−^ cDC subsets that may be important in immune responses.

## Introduction

The dendritic cell (DC) network is essential for the initiation and the regulation of immune responses. Dendritic cells are specialized in Ag capture, processing and presentation on MHC molecules [1]. The interaction of DC with naive T lymphocytes can lead to different forms of immune responses (type 1, type 2 or type 17 responses or tolerance), the outcome of which depends on the type of DC as well as their activation state [2]. Along with their ability to prime naive T lymphocytes and shape the adaptive immune response, DC also play a major role in the activation of innate immune cells including NK and invariant NKT (iNKT) cells. Invariant NKT cells represent an emerging population of “innate-like” immune cells expressing NK lineage receptors and an invariant TCRα chain (Vα14-Jα18 rearrangement in mice and Vα24-Jα18 rearrangement in humans) that pairs with a limited number of Vβ chains. This cell population recognizes exogenous and self (glyco)lipid Ag presented by the CD1d molecule expressed by Ag presenting cells, including DC (for reviews, [3]–[7]). In response to CD1d-restricted lipids such as α-galactosylceramide (α-GalCer), a non-mammalian glycolipid Ag with potent anti-tumor properties [8], iNKT cells rapidly and vigorously produce a wide array of immunomodulatory cytokines including IFN-γ and IL-4. This explosive response leads to downstream activation of DC, NK cells, neutrophils and B and T cells with important outcomes on immune responses and pathologies (for reviews, [3], [7], [9], [10].

Dendritic cells are heterogeneous and can be classified into different subtypes according to their phenotype, tissue distribution and functions. Spleen-resident DC are mainly composed of conventional DC (cDC) that can be further subdivided into distinct subtypes, including CD8α^−^ cDC, encompassing CD4^+^ and CD4^−^ subsets, and CD8α^+^ cDC, expressing or not the CD103 molecule [11], [12]. Over the last decade, several reports pointed out a functional dichotomy between CD8α^−^ cDC, the most numerous cDC subtype in the spleen, and CD8α^+^ cDC. Thus, CD8α^+^ cDC serve as efficient APC for inducing a Th1 response and, through their cross-presenting capacity, for priming CTL response whereas CD8α^−^ cDC preferentially present exogenous Ag to prime CD4^+^ T cells and to induce Th2 responses [1], [13]–[16]. More recently, functional differences within CD8α^+^ cDC subsets have been underlined. Of major importance is the recent demonstration that CD8α^+^CD103^+^ cDC, a subpopulation that localises in the marginal zone of the spleen, is critical for either tolerance induction to cell-associated Ag or cross-priming in response to systemic activation stimuli [17], [18]. Splenic CD8α^−^ cDC subsets (now termed CD4^+^ and CD4^−^ cDC for the sake of simplicity) are closely related phylogenetically although recent transcriptomic and proteomic analyses revealed some differences that may be important for their respective functions [19], [20]. Functional studies aimed at comparing CD4^+^ and CD4^−^ cDC are very limited and sometimes contradictory. Hochrein et *al.* first reported that CD4^−^ cDC are more efficient at producing IL-12 after CD40 ligation or TLR stimulation whereas two other studies reported no major differences in IL-12 production between the two cDC subsets in response to *Leishmania* infection [21]–[23]. Proietto et *al.* also reported that CD4^+^ cDC are the main producers of inflammatory chemokines after TLR activation [24]. The ability of CD4^+^ and CD4^−^ cDC to prime and orientate CD4^+^ T lymphocytes upon sensitization with OVA peptide has been studied in steady-state and stressful conditions. In these systems, both cDC subsets equally primed CD4^+^ T lymphocytes and induced a mixed response. However, it was noticed that the CD4^−^ cDC biased the response towards a more Th1 direction [23], [25], in an IL-12 independent manner [23]. So far, potential differences in the ability of CD4^+^ and CD4^−^ cDC to prime conventional T lymphocytes in response to whole protein remains undetermined. In the present study, we show that after sensitization with OVA peptide or whole OVA, and under steady-state conditions, both CD8α^−^ cDC subsets are comparable in their capacity to prime and direct CD4^+^ and CD8^+^ T cell differentiation. In contrast, when sensitized with the iNKT cell activator α-GalCer, CD4^+^ and CD4^−^ cDC subsets markedly differ, both *in vitro* and *in vivo*, in their ability to activate and/or polarize iNKT cells. Thus, our data reveal a novel functional difference between splenic CD4^+^ and CD4^−^ cDC subsets.

## Results

### CD4^+^ and CD4^−^ cDC are equivalent in their ability to activate CD4^+^ and CD8^+^ T lymphocytes *in vitro*

**Figure 1:**
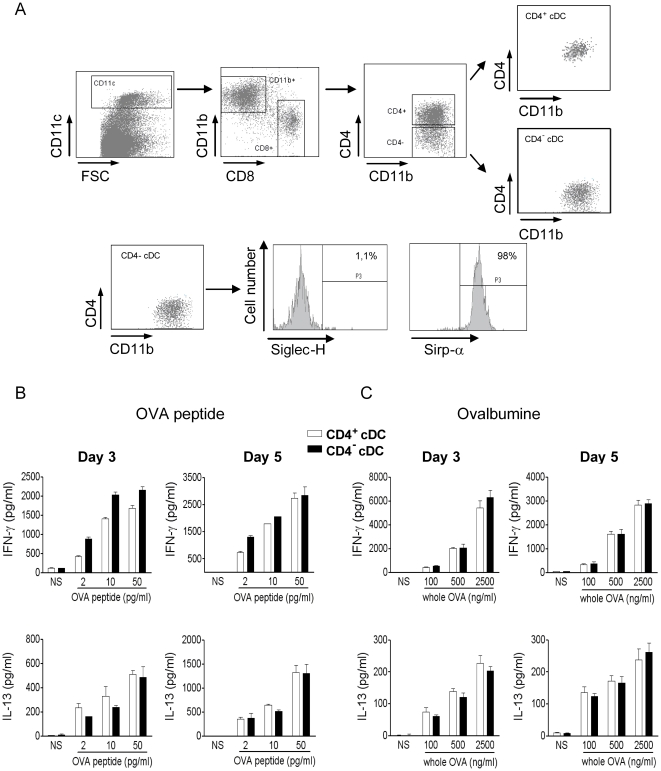
CD4^+^ and CD4^−^ cDC are equivalent in their ability to activate CD4^+^ T lymphocytes. (A) Splenic CD4^+^ and CD4^−^ cDC were sorted on the basis of CD11c, CD11b, CD8 and CD4 expression. The presence of contaminating plasmacytoid DC and CD8α^+^ cDC precursors in the CD4^−^ cDC fraction was evaluated by using anti-Siglec-H and Sirp-α mAbs, respectively. (B, C) Both cDC subsets were sensitized with graded doses of OVA peptide (B) or whole OVA (C) and co-cultured for 3 and 5 days with naive CD4^+^ T cells purified from OT-II mice. The production of IFN-γ and IL-13 was quantified by ELISA. Results represent the mean ± SD of a representative experiment out of two.

There is now evidence that cDC subsets, *i.e.* CD8α^+^ versus CD8α^−^ cDC or CD103^+^ versus CD103^−^ CD8α^+^ cDC, are not equivalent in their Ag presenting functions [13], [16]–[18]. Whether CD8α^−^ cDC subsets (CD4^+^ and CD4^−^) differ in their capacity to present Ag and activate conventional CD4^+^ and CD8^+^ T lymphocytes remains an open question. To address this issue, CD8α^−^ cDC subsets were purified from the spleens of naïve mice on the basis of CD11c, CD4, CD8 as well as CD11b expression. In contrast to previous reports, the CD11b marker was considered in our sorting strategy to limit contaminating cells, principally in the CD4^−^ cDC fraction (Figure 1A). Thus, all CD8α^−^ cDC were CD11b^+^ and the CD4^−^ cDC subset contained very few contaminating cells, such as plasmacytoid DC (Siglec-H^+^) or CD8^+^ cDC precursors (Sirp-α^−^) (Figure 1A). Isolated splenic CD4^+^ and CD4^−^ cDC subsets were then assayed for their ability to prime and direct the differentiation of OVA-specific TCR transgenic T cells (OT-II) in steady-state conditions. As seen in Fig. 1B, CD4^+^ and CD4^−^ cDC loaded with graded doses of the MHC class II-restricted OVA peptide induced a mixed cytokine response in OT-II CD4^+^ T lymphocytes characterized by equivalent levels of secreted IFN-γ and IL-13, whatever the time point analysed. Similarly, loading of CD4^+^ and CD4^−^ cDC with whole OVA resulted in an equivalent release of IFN-γ and IL-13 by OT-II CD4^+^ T lymphocytes (Fig. 1C). IL-4 and IL-5 were undetectable in all culture supernatants. Thus, CD4^+^ and CD4^−^ cDC pulsed with OVA peptide or whole OVA protein equally prime CD4^+^ T lymphocytes to differentiate into a mixed T cell population.

**Figure 2:**
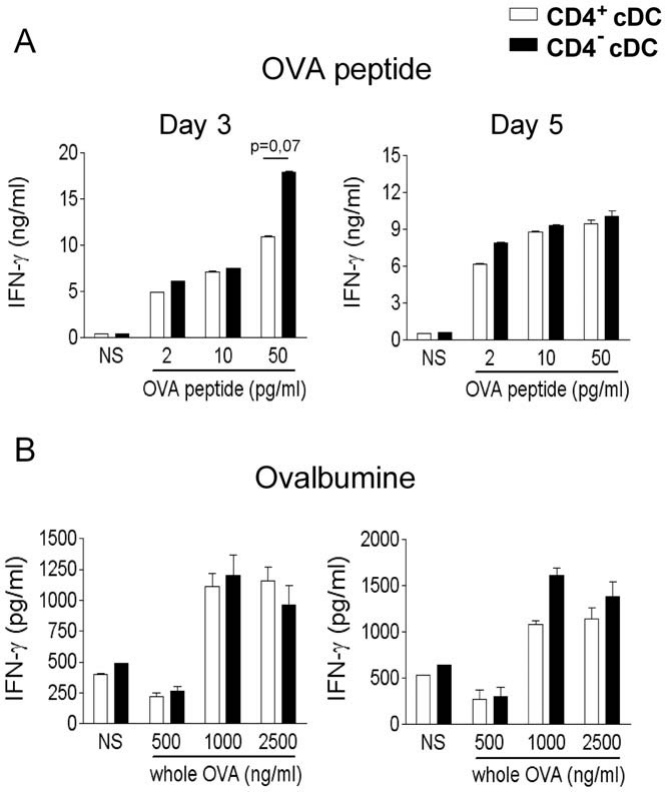
CD4^+^ and CD4^−^ cDC are equivalent in their ability to activate CD8^+^ T lymphocytes. (A, B) CD4^+^ and CD4^−^ cDC subsets were sensitized with graded doses of OVA peptide (A) or whole OVA (B) and co-cultured for 3 and 5 days with naive CD8^+^ T cells purified from OT-I mice. The production of IFN-γ was quantified by ELISA. Results represent the mean ± SD of a representative experiment out of two.

The capacity of CD4^+^ and CD4^−^ cDC to activate CD8^+^ T lymphocytes was next compared. As seen in Fig. 2A, loading of CD4^+^ and CD4^−^ cDC with the MHC class I-restricted OVA peptide SIINFEKL led to a comparable CD8^+^ T cell activation, in terms of IFN-γ production, although for the highest dose, peptide-loaded CD4^−^ cDC induced more IFN-γ but only at day 3. Thus, the two cDC subsets present OVA peptide to CD8^+^ T lymphocytes with the same efficacy leading to an equivalent priming. The cross-presenting ability of both cDC subsets was next assessed using whole OVA (Fig. 2B). Compared to CD8α^+^ cDC [1], [13], [14], [16], CD8α^−^ cDC induced a low, but detectable, production of IFN-γ by OT-I CD4^+^ T lymphocytes. As Fig. 2B shows, the cross-presenting activity of CD4^+^ and CD4^−^ cDC was not statistically different. Collectively, these data indicated that after *in vitro* challenge with peptide or whole protein, CD4^+^ and CD4^−^ cDC have an equivalent capacity to prime naïve CD4^+^ and CD8^+^ T lymphocytes and to induce the differentiation of mixed effector Th populations.

### CD4^+^ and CD4^−^ cDC differ in their capacity to activate iNKT cells *in vitro*

**Figure 3:**
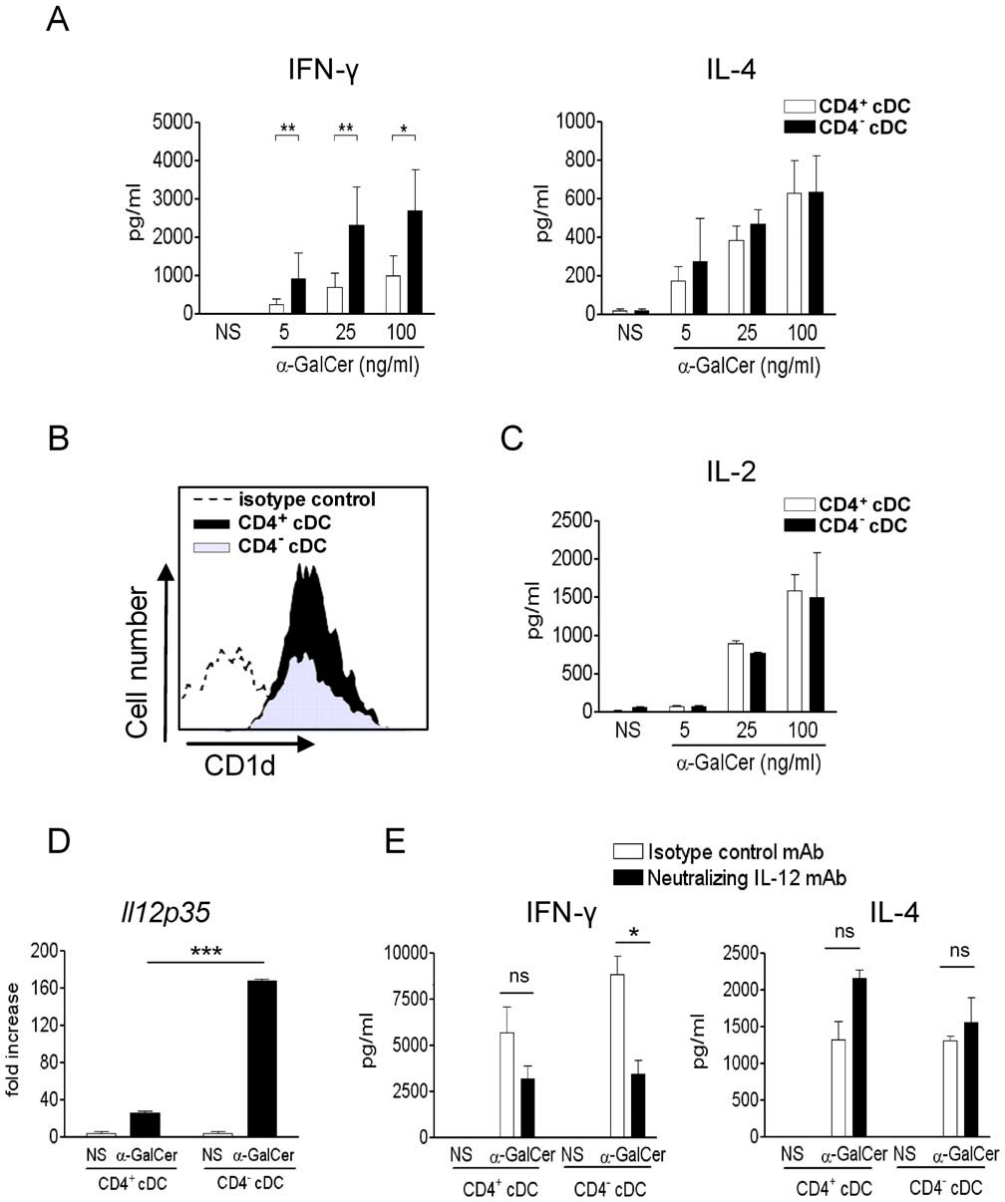
CD4^+^ and CD4^−^ cDC differ *in vitro* in their capacity to activate iNKT cells. (A, C) Sorted CD4^+^ and CD4^−^ cDC were exposed to graded doses of α-GalCer and then co-cultured for 48 h with sorted iNKT cells (A) or with the iNKT cell hybridoma DN32.D3 (C). Cytokine production was quantified by ELISA. Results represent the mean ± SD of 3 (A) or 2 (C) independent experiments. (B) CD1d expression on CD4^+^ and CD4^−^ cDC was assessed by flow cytometry. Of note, the staining with the isotype control was identical on both cDC subsets. For clarity, the isotype control on the CD4^+^, but not CD4^−^, cDC subset is shown. Shown is a representative experiment out of three. (D) Sorted CD4^+^ and CD4^−^ cDC were exposed, or not (medium), to α-GalCer (100 ng/ml) and then co-cultured for 6 h with sorted iNKT cells. RNAs were prepared and IL-12p35 (*Il12p35*) mRNA copy numbers were measured by quantitative RT-PCR. Data are normalized to expression of *Gapdh* and are expressed as fold increase over average gene expression in vehicle-treated cDC. Of note, the basal level of IL-12p40 transcript in *ex vivo* sorted cDC is relatively elevated (Ct: 25–26) (Ct of *gapdh*: 20, Ct of *il12p35*: 31–32). Data represent the mean ± SD (triplicates) of an experiment out of two performed. (E) α-GalCer-loaded cDC subsets were co-cultured for 48 h with sorted iNKT cells in the presence of a neutralizing IL-12 Ab or an isotype control Ab. Shown is a representative experiment (mean ± SD) out of three performed. * p<0.05; ** p<0.01; *** p<0.001.

Dendritic cells are particularly well equipped to promote rapid and potent cytokine release by iNKT cells [26]–[32]. We first compared the capacity of CD4^+^ and CD4^−^ cDC to activate iNKT cells *in vitro*. To this end, the two cDC subsets were loaded with the prototypical lipid Ag α-GalCer, washed and then exposed to primary iNKT cells. As depicted in Fig. 3A, α-GalCer-loaded CD4^−^ and CD4^+^ cDC promoted the production of both IFN-γ and IL-4 by FACS-sorted iNKT cells in a dose-dependent manner. Interestingly, whilst both cDC subsets induced an equivalent secretion of IL-4 by iNKT cells, CD4^−^ cDC triggered a significantly higher production of IFN-γ by iNKT cells relative to their CD4^+^ counterparts. This effect was observed whatever the dose of α-GalCer. We hypothesized that this difference could be due to a differential expression of the CD1d molecule on the DC surface. However, flow cytometry analysis revealed an equivalent level of CD1d expression between CD4^−^ (MFI of 8795±543) and CD4^+^ (MFI of 9710±503) cDC subsets in steady-state conditions (Fig. 3B) as well as after *in vitro* α-GalCer loading (data not shown). Moreover, the DN32.D3 hybridoma, the activation of which is solely due to CD1d/TCR interactions, was activated to a similar extent by both CD4^+^ and CD4^−^ cDC (Fig. 3C). These data suggest that the enhanced ability of CD4^−^ cDC to polarize primary iNKT cells towards a Th1 direction is probably due to co-factors produced by the former. We investigated the possibility that IL-12 could be responsible for this phenomenon. Interleukin-12p40 and p70 proteins were not detected in our co-culture system, a phenomenon that could be explained by their rapid capture. In line with this hypothesis, and relative to the control, an induction of IL-12p35 (but not IL-12p40, not shown) transcripts was detected in α-GalCer loaded cDC/iNKT cell co-culture (Fig. 3D). Of interest, the fold induction of IL-12p35 mRNA synthesis (∼160 fold) was more elevated in CD4^−^ cDC compared to CD4^+^ cDC (∼20 fold). These data suggest that bioactive IL-12 might be involved in the higher ability of α-GalCer pulsed CD4^−^ cDC to induce IFN-γ by primary iNKT cells. To confirm this hypothesis, a neutralizing anti-IL-12 Ab was added during the co-culture. As already reported [33], [34], in this system, IFN-γ production by iNKT cells was not fully dependent on IL-12 production by cDC (Fig. 3E). However, the enhanced capacity of CD4^−^ cDC to promote IFN-γ production by iNKT cells was totally dependent on IL-12.

### CD4^+^ and CD4^−^ cDC loaded *in vivo* with α-GalCer activate iNKT cells differently

**Figure 4:**
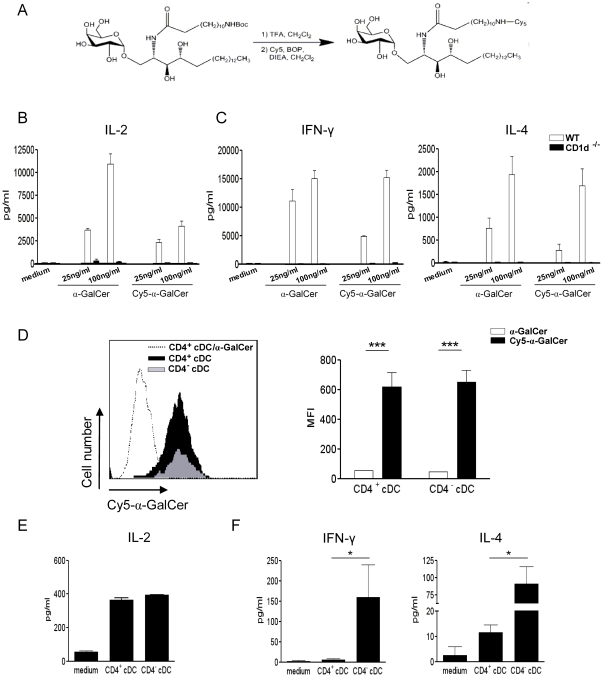
CD4^+^ and CD4^−^ cDC equally capture α-GalCer *in vivo* but differ in their ability to activate iNKT cells. (A) Representation of Cy5-conjugated α-GalCer synthesis. (B, C) Bone marrow-derived DC (10^5^/well) from WT and CD1d^−/−^ mice were loaded with unconjugated or Cy5-conjugated α-GalCer (25 and 100 ng/ml) and then co-cultured with either the iNKT cell hybridoma DN32.D3 (10^5^/well) for 24 h (B) or with sorted primary iNKT cells (10^5^/well) for 48 h (C). Cytokine production was quantified by ELISA. (D) Recipient mice were i.v. injected with Cy5-conjugated (20 µg), or unconjugated as a control (dotted line), α-GalCer and then Cy5 incorporation by CD4^+^ (black) and CD4^−^ cDC (grey) was analyzed by flow cytometry 2 h later. Of note, both cDC subsets had similar profiles using unconjugated α-GalCer. For clarity, we only show the FACS profile for the CD4^+^ cDC subset. Shown are a representative histogram (left panel) and the MFI ± SD (right panel) of three independent experiments. (E, F) Mice were i.v. injected with α-GalCer (2 µg), cDC subsets were sorted 2 h later and co-cultured with the iNKT cell hybridoma DN32.D3 (E) or with sorted iNKT cells (F). Cytokine production was quantified by ELISA. Shown is a representative experiment out of three performed. * p<0.05; *** p<0.001.

We then compared the ability of CD4^+^ and CD4^−^ cDC, loaded *in vivo* with α-GalCer, to activate iNKT cells. Before this, we verified that both cDC subsets can take up α-GalCer *in vivo* after intravenous injection. To this end, Cy5 conjugated to α-GalCer was synthesized, as shown schematically in Fig. 4A, and tested for its *in vitro* iNKT cell activating property. As revealed in Fig. 4B, *in vitro* exposure of bone-marrow derived DC to increasing doses of Cy5-α-GalCer promoted IL-2 production by DN32.D3 hybridoma cells. This effect was dependent on CD1d expression by DC. Cy5-α-GalCer also induced, in a CD1d-dependent fashion, IFN-γ and IL-4 release by primary iNKT cells (Fig. 4C). We next compared the *in vivo* incorporation rate of α-GalCer in cDC subsets. As shown in Fig. 4D, 2 h after Cy5-α-GalCer administration, both CD4^+^ and CD4^−^ cDC labelled positively and to a similar extent. Of note, Cy5-conjugated α-GalCer was weakly incorporated by both cDC subsets 45 min after injection (data not shown). The *ex vivo* iNKT cell-activating properties of CD4^+^ and CD4^−^ cDC were then compared. To this end, cDC subsets were sorted from α-GalCer-inoculated mice (2 h) and co-cultured with iNKT cells. At this time point, cDC subsets equally expressed the CD1d molecule and exhibited no sign of maturation, as assessed by flow cytometry (data not shown). As shown in Fig. 4E, *in vivo* α-GalCer sensitized CD4^+^ and CD4^−^ cDC activated the DN32.D3 hybridoma to the same extent although in a less efficient manner compared to *in vitro* α-GalCer sensitized cDC (Fig. 3C). These data are in line with the above results showing no differences in the incorporation rate of α-GalCer *in vivo* between the two cDC subsets. In contrast, when primary cell-sorted iNKT cells were used, major differences were observed (Fig. 4F). Indeed, CD4^−^ cDC promoted IFN-γ and IL-4 release by primary cell-sorted iNKT cells while CD4^+^ cDC only promoted a low level of IL-4 secretion.

## Discussion

Very few studies have been devoted to investigate the respective roles of CD4^−^ and CD4^+^ cDC subsets in immune responses. In the current study, we showed for the first time that upon peptide and whole protein challenge, splenic CD4^−^ and CD4^+^ cDC equally prime CD4^+^ and CD8^+^ conventional T lymphocytes *in vitro* and that under steady-state conditions, they have a similar capacity to induce the differentiation of mixed effector Th populations. In marked contrast, CD4^−^ and CD4^+^ cDC differ in their ability to stimulate iNKT cells.

In this study, we compared the activating properties of ex *vivo*-isolated CD4^−^ and CD4^+^ cDC on conventional T lymphocytes (priming and polarization) as well as on iNKT cells (primary stimulation), a population of non-conventional T lymphocytes. Even under stringent sorting conditions, the functions attributed to cDC subsets may be biased by minor contaminant populations. In contrast to previous reports [19]–[25], a particular attention was paid to the elimination of potential contaminating cells such as plasmacytoid DC and Sirp-α-negative CD8^+^ cDC precursors in the CD4^−^ cDC preparation. We first compared the capacity of CD4^−^ and CD4^+^ cDC to prime and orientate naïve T cells *in vitro* using OVA peptide or whole OVA protein, as a model of study. In agreement with [23], [25], we showed that, when pulsed with OVA peptide, splenic CD4^−^ and CD4^+^ cDC equally prime naïve CD4^+^ conventional T cells *in vitro*. Similarly, no differences between the two cDC subsets were found in response to whole OVA. This novel observation indicated that, at least for OVA and in steady-state conditions, CD4^+^ and CD4^−^ cDC share similar endocytic properties as well as an equivalent ability to trim Ag and present peptide fragments by the MHC class II molecule. Of note, MHC class II molecules were equally expressed on both cDC subsets (not shown). Our data also suggested no differences in the Th directing potential of CD4^+^ and CD4^−^ cDC in our experimental conditions. This finding is in line with [25], but not with [23] who showed that CD4^−^ cDC rather favour Th1 polarisation relative to CD4^+^ cDC. When exposed to a MHC class I-restricted peptide, no major differences in priming activity of CD4^+^ and CD4^−^ cDC was noticed. Finally, in accordance with the poor cross-presenting capacity of CD8α^−^ cDC [1], [13], [14], CD4^+^ and CD4^−^ cDC pulsed with whole OVA modestly activated OT-I CD8^+^ T lymphocytes. In these settings, CD4^+^ and CD4^−^ cDC activate OVA-specific CD8^+^ T lymphocytes to a similar extent. Thus, our study clearly show that, under steady-state conditions, highly purified CD4^+^ and CD4^−^ cDC prime and orientate CD4^+^ and CD8^+^ T lymphocytes with the same efficacy. CD8α^−^ cDC are particularly well equipped with innate (microbial) detectors [19], [20]. Whether or not functional differences in terms of both stimulatory and regulatory properties occur between CD4^+^ and CD4^−^ cDC during stressful conditions (*i.e.* in the context of infection) is unknown at present. Previous studies suggested that CD4^−^ cDC activated *in vivo* during *Leishmania donovani* infection or *in vitro* upon TLR activation are more prone to induce a Th1 response than their CD4^+^ cDC counterparts [23], [25]. Thus, CD4^−^ and CD4^+^ cDC may play different roles in immune responses in the context of exogenous (microbial) stimulation.

We next assessed whether CD4^−^ and CD4^+^ cDC promote identical primary stimulation of iNKT cells, an “innate-like” immune cell population that reacts to lipid Ag. Previous findings established that cDC are particularly efficient at activating iNKT cells *in vivo* [26], [28] and that, in turn, iNKT cells strongly contribute to DC maturation and functions [35]. Studies from Farrand et *al.* and Simkins et *al.* showed that, after α-GalCer administration, CD8α^+^ CD103^+^ (CD207^+^) cDC are dispensable for the primary activation of iNKT cells but play a key role in the by-stander activation of immune cells, which occurs subsequently to primary iNKT cell activation [17], [36]. These data suggested a role for other APC, including CD8α^−^ cDC, in the primary activation of iNKT cells. No studies have so far evaluated the respective role of CD4^−^ and CD4^+^ cDC in this setting. Analysis of CD1d expression revealed no differences between CD4^−^ and CD4^+^ cDC freshly sorted from naïve animals (Fig. 3B) or *in vivo* early after α-GalCer inoculation (data not shown). Moreover, *in vitro* as well as *in vivo* sensitization with α-GalCer triggered an activation of the iNKT cell hybridoma DN32.D3, as assessed by IL-2 production. These data, and the fact that α-GalCer is incorporated with the same kinetics and efficacy by both cDC subsets *in vivo* (Fig. 4D), indicated that CD4^−^ and CD4^+^ cDC display no differences in their ability to trigger TCR-dependent activation of iNKT cells. α-GalCer can directly bind to cell-surface CD1d but a large proportion of it is internalised and loaded on CD1d in endosomes [37], [38]. This suggests that acquisition and uptake of free α-GalCer in cDC as well as mechanisms regulating the CD1d Ag presentation pathway are not profoundly different between the two cDC subsets.

Activation of primary iNKT cells, unlike iNKT hybridomas, not only depends on CD1d/Ag mediated TCR triggering, but also on co-factors produced by DC. This complex interplay between DC and iNKT cells not only modulates the strength of the iNKT cell response but also the nature of released cytokines. We found major differences between CD4^−^ and CD4^+^ cDC in their capacity to promote activation of primary iNKT cells, as judged by cytokine release. *In vitro*, we consistently observed that CD4^−^ cDC induced more IFN-γ secretion by iNKT cells than their CD4^+^ cDC counterparts. Previous reports have shown that, following initial DC/iNKT cell contact, production of bioactive IL-12 by α-GalCer loaded DC is necessary for optimal production of IFN-γ by iNKT cells [33], [34]. On the other hand, under steady-state conditions, IL-12 is not required for primary activation of conventional T lymphocytes [25], [39], [40]. Of note, there is only a moderate enhancement (∼2 to 3 fold, for both cDC subsets) of IL12p35 mRNA levels in this condition (data not shown). However, in contrast to conventional T lymphocytes, our data show that the enhanced capacity of CD4^−^ cDC (compared to CD4^+^ cDC) to promote IFN-γ production by iNKT cells is IL-12 dependent. This result is in line with the higher capacity of CD4^−^ cDC to release bioactive IL-12 under stressed conditions [22]. As a whole, as is the case after TLR stimulation [22], our data support the notion that CD4^−^ cDC are stronger producers of bioactive IL-12 after iNKT cell-mediated cDC maturation. The *ex vivo* stimulatory activity of CD4^−^ and CD4^+^ cDC on primary iNKT cells was next compared. Despite an identical α-GalCer uptake rate *in vivo* and a similar level of cell surface CD1d relative to CD4^−^ cDC, CD4^+^ cDC failed to trigger IFN-γ release and promoted a low amount of IL-4 by primary iNKT cells. However, both cDC subsets were able to activate the iNKT hybridoma DN32.D3, the activation of which requires less cell surface CD1d/α-GalCer complex than that of primary iNKT cells. The inferior ability of CD4^+^ cDC to promote IFN-γ release is in agreement with our *in vitro* data and can be explained by their weaker IL-12 production following initial contact with iNKT cells. The lower amplitude of primary iNKT cell activation following contact with *in vivo* loaded cDC, relative to *in vitro* loaded cDC, certainly amplifies this phenomenon. Moreover, it is possible that the expression of other co-stimulatory factors by *in vivo* α-GalCer loaded CD4^+^ cDC is insufficient to promote optimal activation of iNKT cells (IL-4) in our experimental settings [41], [42], [43], [44]. It is also possible that expression of inhibitory molecules by CD4^+^ cDC may have altered the threshold for activation of primary iNKT cells.

To conclude, our data show that, under steady-state conditions, CD4^−^ and CD4^+^ cDC equally prime and orientate conventional T lymphocytes *in vitro*. This suggests that targeting vaccine Ag to either DC subset would be without potential benefit. In contrast, our study shows for the first time, that CD4^−^ cDC are more potent in stimulating iNKT cells, at least in response to the canonical iNKT cell agonist α-GalCer. It remains to define whether CD4^−^ and CD4^+^ cDC display a similar behaviour in response to α-GalCer-based analogues or to more physiological (self lipids) ligands. The physiological relevance of this novel finding on iNKT cell-mediated immune responses awaits further studies.

## Materials and Methods

### Mice

Six- to 8-wk-old male wild type C57BL/6 mice were purchased from Janvier (Le Genest-St-Isle, France). 8-wk-old male OT-I or OT-II mice were purchased from Iffa Credo (St. Germain sur l'Arbresle, France). The generation of CD1d^−/−^ mice has been already described [45]. Mice were bred in our own facility in pathogen free conditions. Animals were handled and housed in accordance with the guidelines of the Pasteur institute Animal Care and Use Committee. All the experiments were performed after approval by the ethics committee for animal experimentation from the Nord–Pas de Calais Region (Agreement N°: 59-350163).

### Reagents and Abs

α-GalCer was purchased from Axxora Life Sciences (Coger S.A., Paris, France). The MHC class I- and MCH class II-restricted OVA peptides, respectively SIINFEKL and ISQAVHAAHAEINEAGR were synthesized by the Institut de Biologie et Chimie des Protéines (Lyon, France). OVA was purchased from Sigma-Aldrich (Sigma, St Quentin-Fallavier, France). APC-conjugated monoclonal Abs against mouse CD5, CD11c, PE-conjugated anti-NK1.1, -CD11b, -CD4, -Sirp-α, FITC-conjugated anti-CD8, Siglec-H, PercPCy5.5-conjugated anti-CD4, -CD11b, biotin-conjugated -CD1d, -CD86 and PE-Cy7-conjugated anti-CD8, streptavidin and isotype controls were purchased from BD Pharmingen (Le Pont de Claix, France). Biotin-conjugated anti-CD40 were purchased from eBioscience (Montrouge, France). The neutralizing goat IgG directed against mouse IL-12 was from R&D systems (Abingdon, UK) and the isotype control Ab from Sigma. Cyanine (Cy)5-conjugated α-GalCer was synthesized according to Kamijuku *et al.* [46], except that Cy3-NHS was replaced by Cy5-COOH on the amino-terminal position of the α-GalCer sphingosine moiety (Fig. 3A). Briefly, the primary amine compound was stirred in dichloromethane in the presence of N,N-Diisopropylethylamine and benzotriazole-1-yl-oxy-tris-(dimethylamino)-phosphonium hexafluorophosphate with Cy5 to provide α-GalCer-Cy5.

### Purification of splenic CD4^−^ and CD4^+^ cDC subsets

For cDC cell sorting, spleens were treated with type VIII collagenase (1 mg/ml) (Sigma) at 37°C for 20 min and then disrupted in PBS supplemented with 2% FCS. After washes, red blood cells were removed using lysis buffer (Sigma)). For cDC cell sorting, spleen cells were labeled with APC-conjugated anti-CD11c, PE-conjugated anti-CD11b, PerCpCy5.5-conjugated anti-CD4 and FITC-conjugated anti-CD8 mAbs. After cell surface labeling and washing, cells were electronically sorted using a FACSAria (Becton Dickinson, MD, USA). Sorted splenic cDC subsets were at least 98% pure.

### Purification of iNKT cells

Perfused livers were minced into small pieces and incubated with type VIII collagenase (1 mg/ml) and DNase I (1 mg/ml) (Sigma) at 37°C for 20 min. The liver pieces were then homogenized and NKT cells were enriched by centrifugation in a 36%–72% Percoll gradient. Liver mononuclear cells were labeled with APC-conjugated anti-CD5 and PE-conjugated anti-NK1.1 mAbs and cells were sorted as described [47]. CD5^+^ NK1.1^+^ cell purity after sorting was consistently >98%. Sorted CD5^+^ NK1.1^+^ cells contain ∼90% iNKT cells as assessed by PBS57-loaded CD1d tetramer and TCRβ staining.

### FACS analysis

Briefly, 2×10^6^ cells/well were plated in 96-well plates, resuspended in 50 µl of the appropriate combination of Abs (allowing cDC subset identification) and incubated on ice for 30 min. After washes, biotin-conjugated anti-CD1d, -CD86 or -CD40 Ab or isotype control were added for another 30 min. Then, PE-Cy7-conjugated streptavidin was added for 20 min. After the last wash, cells were fixed in 1% paraformaldehyde in PBS and were analyzed on a LSR Fortessa (Becton Dickinson). Data were then analyzed using FlowJo software (Treestar, OR, USA).

### Assessment of IL-12 gene expression by quantitative RT-PCR

Total RNA from DC/iNKT cell co-culture was extracted and cDNA was synthesized by classical procedures. Quantitative RT-PCR was carried out in an ABI 7500 Thermocycler (Applied Biosystems, Foster City, CA) using 0.5 µM of specific primers and QuantiTect SYBR Green PCR Master Mix (Qiagen). Primers specific for *gapdh* 5′-TGCCCAGAACATCATCCCTG-3′ and 5′-TCAGATCCACGACGGACACA-3′, *Il12p35* 5′-CACGCTACCTCCTCTTTTTG-3′ and 5′-CAGCAGTGCAGGAATAATGTT-3′, *Il12p40* 5′-GACCCTGCCCATTGAACTGGC-3′ and 5′-CAACGTTGCATCCTAGGATCG-3′, were designed by the Primer Express Program (Applied Biosystems) and used for amplification in triplicate assays. PCR amplification of *gapdh* was performed to control for sample loading and to allow normalization between samples. ΔCt values were obtained by deducting the raw cycle threshold (Ct values) obtained for *gapdh* mRNA, the internal standard, from the Ct values obtained for investigated genes. For graphical representation, data are expressed as fold mRNA level increase compared to the expression level in vehicle-treated DC/iNKT cell co-culture.

### Dendritic cells and iNKT co-cultures

Bone-marrow DC were prepared as described in [48]. Cell-sorted cDC subsets (3×10^4^/well, 96-well plate) were pulsed with OVA peptides for 2 h or whole OVA for 6 h, washed and co-cultured with 1.5×10^5^ naïve CD4^+^ or CD8^+^ T lymphocytes purified from the spleens of OT-II and OT-I mice, respectively. After 3 and 5 days, culture supernatants were collected and cytokine production was analysed by ELISA (R&D Systems and eBiosciences). For cDC and iNKT cell co-cultures, cDC subsets (10^4^ to 10^5^/well) were pulsed with graded doses of α-GalCer for 4 h, washed, and co-cultured for 48 h with hepatic CD5^+^ NK1.1^+^ cells (5×10^4^ cells/well) or the iNKT cell hybridoma DN32.D3 [49] (10^5^ cells/well). In some experiments, α-GalCer-loaded cDC subsets were co-cultured with sorted iNKT cells in the presence of a neutralizing IL-12 Ab or an isotype control Ab (10 µg/ml). To study the *ex vivo* stimulatory capacity of cDC subsets, mice were i.v. injected with 2 µg of α-GalCer, cDC subsets were sorted 2 h later and co-cultured (7×10^4^ cells/well) with 10^5^ iNKT cell hybridoma for 24 h or with 10^5^ sorted hepatic iNKT cells for 48 h. Cytokine production was measured in the culture supernatants by ELISA.

### Measurement of Cy5-α-GalCer incorporation by splenic CD4^−^ and CD4^+^ cDC subsets

Mice were i.v. injected with Cy5-conjugated α-GalCer (20 µg) and 45 min or 2 h later, spleen cells were labelled with appropriate fluorescent Abs to identify splenic cDC subsets. Spleen cells were acquired on the LSR Fortessa and data analyzed using the FlowJo software.

### Statistics

Results are expressed as the mean ± SD. The statistical significance of differences between experimental groups was calculated by an unpaired Student's t test or an ANOVA1 with a Bonferroni post test (GraphPad Prism 4 software, San Diego, CA). Results with a *p* value of less than 0.05 were considered significant.
